# Phenotypic MicroRNA Microarrays

**DOI:** 10.3390/microarrays2020063

**Published:** 2013-04-03

**Authors:** Yong-Jun Kwon, Jin Yeong Heo, Hi Chul Kim, Jin Yeop Kim, Michel Liuzzi, Veronica Soloveva

**Affiliations:** Institut Pasteur Korea, IP-Korea, 696 Sampyeong-dong, Bundang-gu, Seongnam-si, Gyeonggi-do 463-400, Korea; E-Mails: yjkwon@ip-korea.org (Y.-J.K.); maple3644@ip-korea.org (J.Y.H.); island7boy@ip-korea.org (H.C.K.); jykim@ip-korea.org (J.Y.K.); michel.liuzzi@ip-korea.org (M.L.)

**Keywords:** microRNA, siRNA, phenotypic screen

## Abstract

Microarray technology has become a very popular approach in cases where multiple experiments need to be conducted repeatedly or done with a variety of samples. In our lab, we are applying our high density spots microarray approach to microscopy visualization of the effects of transiently introduced siRNA or cDNA on cellular morphology or phenotype. In this publication, we are discussing the possibility of using this micro-scale high throughput process to study the role of microRNAs in the biology of selected cellular models. After reverse-transfection of microRNAs and siRNA, the cellular phenotype generated by microRNAs regulated NF-κB expression comparably to the siRNA. The ability to print microRNA molecules for reverse transfection into cells is opening up the wide horizon for the phenotypic high content screening of microRNA libraries using cellular disease models.

## 1. Introduction

Two key events in biological sciences happened a decade ago around the year 2000: first, the sequence of the human genome was completed and published by Celera Co. and NIH, and second, the discovery of RNA interference was reported, where small double-stranded oligonucleotides mediate post-transcriptional gene silencing in a broad variety of organisms [1,2,3]. Since then, the new wave of technological applications has facilitated research and provided tools for basic research studies and drug discovery. Double-stranded RNA was found to mediate sequence-specific, post-transcriptional knock-down of the mRNA in cells and tissues of all origins of plants, insects and animals. RNA-mediated interference (RNAi) became useful in the analysis of functional genes and their roles in biological phenotypes in mammalian and particular human cells [3,4]. With the development of synthetic siRNAs and shRNAs, the RNA-interference methods were adopted for systematic and methodical screening approaches in studies of diverse biological processes, including mechanisms of disease pathogenesis [5,6,7]. 

The miRNAs (microRNA, miR) in contrast to the synthetic inhibitory RNAs, like siRNA and shRNA, are encoded by the genome and function as endogenous regulatory factors for both protein coding genes and non-coding RNAs. The expression of specific miRNAs in mammalian tissue was found to negatively correlate with the level of the mRNAs for genes whose 3'UTRs are targeted by miRNA [8]. Those and similar observations lead to the conclusion that miRNAs can control tissue- and disease-specific gene expression, mostly at the mRNA level. In brief, miRNA can induce two processes: the more common pathway involves promoting mRNA degradation by creating a perfect duplex and, thus, inducing duplex cleavage by the RNA-induced silencing complex (RISC) complex; the other process involves inhibiting mRNA translation by the formation of imperfect duplexes [8,9,10,11,12]. With the current knowledge of RNAi, the miRNA molecules can be engineered to mimic natural miRNAs and used to control the expression of genes of interest [13]. However, altogether, there are hundreds and thousands of natural miRNAs and synthetic miRNA mimics that could be involved in the regulation of at least 60% of genes in the human genome. The study of those molecules and of their function requires a robust methodology with a very good throughput. The hybridization techniques, like LNA or Q-PCR, were mostly used as a screening approach to search for miRNAs involved in the control of different cellular phenotypes or disease phenotypes of interest [14,15]. That allowed scientists to quantify overexpression of miRNAs in those cellular or tissue models, but did not reveal the function of each of the selected molecules.

Recently, several screens were conducted to test miRNA collections for their specific effects on the expression of co-transfected reporter gene constructs [16,17,18]. However, the non-physiological overexpression of reporter genes cannot reflect correctly the complex mechanisms that are involved in regulation of post-transcriptional events. In this work, we are developing a phenotypic screening approach that would allow monitoring of the functional response of cells transfected with different RNAi molecules.

High density spot microarrays became popular after 1995, when P. Brown with his group [19] created the first bimolecular printing solution for DNA microarrays. The microarray approach is useful when there is a need for repetitive analyses of different samples with a large collection of the probes, like polypeptides, mRNA (cDNAs), *etc.* [20,21]. The first application of microarrays that introduced genetic material into a monolayers of cultured cells was first suggested and demonstrated by Ziauddin, J. and Sabatini, M. in 2001 [22]. Even though this screen tested only 192 cDNA constructs, it clearly demonstrated the advantages of throughput and the flexibility of the microarray format for cell-based screening of a bio-molecule collection. The feasibility of using a phenotypic approach instead of a DNA or RNA hybridization method opened up a broad spectrum of applications for cellular assays. It also highlighted the conceptual idea that the cellular phenotypic response could be used in an automated high throughput mode with reproducible precision and accuracy, ensuring identification of new genes [23,24].

To develop the microarray application for over-expression of microRNAs, we selected the regulation of endogenous NF-κB expression in cancer cells. Nuclear factor kappa-light-chain-enhancer of activated B-cells (NF-κB) is a protein complex that controls DNA transcription and regulates diverse signaling processes in many animal cells. Incorrect regulation of NF-κB has been associated with cancer, inflammatory and autoimmune diseases, due to the control of cell proliferation, differentiation and survival [25,26]. There are five members in the NF-κB family. Proteins from class I NF-κB1 or p50 and NF-κB2 or p52 and class II RelA, RelB and c-Rel can form homo- or hetero-dimers to create complexes that are able to control gene transcription [27]. Due to the critical role of NF-κB in cellular functions, the regulation of its expression and activation happens at several different levels. One pathway controls the existing level of inactive NF-κB dimers by its association with the inhibitory protein complex (IkB). The degradation of IkB frees NF-κB for activation and transport into the nucleus. The other pathway is more complex and includes post-translational modifications of different members of the NF-κB family, as well as IkB and related signaling cascades (review Oeckinghaus and Ghosh 2009 [28]). 

MicroRNAs have been shown to be involved in cell differentiation, immune response and tumor development and metastasis progression [29,30,31,32]. It is not surprising that there have been several studies trying to establish connections between NF-κB signaling pathways and microRNA functions and, in particular, tumor development and progression [16,33,34,35,36]. Several miRNAs, like miR-146, miR-155, miR-181b, miR-21 and miR-301a, are involved in NF-κB activation, and at the same time, they play a significant role in tumorigenesis [31]. In line with bioinformatic predictions, the co-transfection of reporter genes and microRNA from the miR-520/373 family reduced expression of the reporter-gene associated with RelA 3'UTR [16,35,37]. In our work, we are using the regulation of NF-κB (RelA) expression as a model to develop a new microarray-based approach that can facilitate the use of phenotypic analysis of cellular responses in the search for new critical modulators within a collection or library of microRNAs or microRNA mimic molecules [13].

## 2. Experimental Methods

### 2.1. Chemicals and Cell Culture

All fine chemicals were purchased from Sigma-Aldrich. DRAQ5 (Cat#: DR50050) was from BioStatus (Shepshed, UK). The OTP (On-TARGET *plus*) version of siRNA duplexes—RelA siRNA smart pool, L-003533-00-0005, non-targeted siRNA #1, D-001810-01-05 and human miRIDIAN microRNA: has-miR-373 mimic, C-300680-03-0005, has-miR-520c-3p mimic C-300803-05-0005 and microRNA mimic negative control #1, CN-001000-01-05—were purchased from Dharmacon (Lafayette, CO, USA). Primary antibodies, NF-κB p65(# SC-109), were from Santa Cruz Biotechnology and fluorescent secondary antibodies, Alexa Fluor^®^ 488 donkey anti-rabbit IgG (H+L), MOP-A-21206, from Molecular Probes/Invitrogen (Carlsbad, CA, USA). 

Hela cells (ATCC, Manassas, VA, USA) were cultivated in high-glucose Dulbecco’s Modified Eagle Medium (DMEM) (Invitrogen, Carlsbad, CA, USA) supplemented with glutamax, 110 mg/mL sodium pyruvate, 10% fetal calf serum (Gibco, Carlsbad, CA, USA) and 1% penicillin streptomycin (Invitrogen Carlsbad, CA, USA). For siRNA forward transfection, Hela cells were seeded at 1.5 × 10^4^ cells/well in 96-well plates and cultured in growth medium without antibiotics for 16 h before transfection. For reverse transfection on arrays, cells were seeded at 2 × 10^6^ cells/array and cultured in OptiMEM medium supplemented with 5% fetal calf serum (Gibco) and 1% penicillin streptomycin (Invitrogen) for 48 h.

### 2.2. miRNA and siRNA Transient Transfection

Hela cells were trypsinized one day before transfection, diluted in fresh DMEM high glucose medium supplemented with 5% FBS without antibiotics and transferred to 96-well plates (Greiner, Washington, DC, USA). 5,000 Hela cells were seeded per well and cultivated for 16 h. Transient transfection of siRNAs was carried out using DharmaFECT 2 (Dharmacon, Thermofisher, West Lafayette, IN, USA). For each well, 9.9 µL of serum-free DMEM and 0.1 µL of DharmaFECT 2 were pre-incubated for 5 min at room temperature. At the same time, 5 µL of serum-free DMEM were mixed with 5 µL of each siRNA (1 µM) and also incubated for 5 min at room temperature. The two mixtures were combined and incubated for 20 min at room temperature for complex formation. After addition of 80 µL of complete DMEM medium to the mixture, the entire solution was added to the cells in each well, resulting in a final concentration of 50 nM for the siRNAs. After transfection, cells were incubated for 48 h to allow gene silencing to occur. 

### 2.3. Phenotypic NF-κB Assay and Data Analysis

To develop the NF-κB detection assay, cells were transfected with siRNAs for 48–72 h. After incubation, the cells were washed twice with phosphate-buffered saline (PBS), fixed for 10 min with 4% (w/v) paraformaldehyde in PBS, washed again with PBS and permeabilized with 0.1% (v/v) Triton X 100 in PBS for 10 min. Permeabilized cells were washed in PBS and incubated with a 1:200 dilution of anti-p65 antibody (Rel A, #SC-109, Santa Cruz, CA, USA) in 10% (v/v) goat serum PBS overnight at 4 °C. Cells were washed 3 times with PBS for 10 min on an orbital rotator and treated with Alexa 488 goat anti-rabbit secondary antibody (1:1,000) (Molecular Probes) for 60 min at room temperature. Cells were washed 3 times for 10 min with PBS on an orbital rotator before the addition of 5 μM DRAQ5 in PBS and incubated for 10 min at room temperature. The images of the cells on the plates were acquired with a 20× objective using an ImageXpress Ultra point scanning confocal microscope (Molecular Devices, Sunnyvale, CA, USA). Images were acquired at 488 nm (fluorescein isothiocyanate (FITC)) to detect RelA expression, and at 635 nm, to record nucleus staining with DRAQ5. Quantification of RelA silencing was performed using MetaXpress software (Molecular Devices). Cells were identified and counted using the nucleus mask. The intensity of the RelA signal was a threshold to exclude the population of cells expressing low levels of NF-κB. The data were normalized as a ratio of cells with an intensity of FITC/pixel above the threshold to the total amount of cells detected in the image at 635 nm. The ratio value of cells in wells with non-target control transfection was used as 100%. The numbers of cells that show the RelA signal higher than the threshold were converted in the % of the total cell number in that image or the percent of expressing cells. Statistical analysis of the data was done using GraphPad Prism.

### 2.4. RNA Extraction and Real-Time PCR

Total RNA was extracted from Hela (up to 10^6^) cells using Trizol reagent (Invitrogen). Target RNA was reverse transcribed using the Moloney murine leukemia virus (MMLV) reverse transcriptase enzyme (Promega). In the first step, 5 μM Oligo (dT) 16 was added to 0.5–1 μg of total RNA and annealed at 70 °C for 10 min. Then, 100 U MMLV reverse transcriptase was added in the presence of 50 mM Tris-HCl, pH 8.3, 75 mM KCl, 3 mM MgCl_2_ and 5 mM unlabeled deoxynucleotides (dNTPs) and incubated at 37 °C for 60 min. For each experiment, RT (reverse transcriptase)-minus controls were included to provide a negative control for subsequent PCR reactions. For PCR amplification, a maximum of 1 μL of cDNA was used per 50 μL PCR. Larger amounts were avoided, because they might significantly inhibit PCR amplification. To minimize variations in reverse transcriptase efficiency, all samples from a single experiment were reverse transcribed simultaneously. 

Real-time PCR was performed with the SYBR Premix Ex Taq II (TaKaRa Bio Inc., Shiga, Japan) and MJ Research PTC-200 Thermo Cycler (BioRad, Hercules, CA, USA). Amplification was done in a 20 µL final volume containing 1–5 μL of template cDNA, 10 μL of SYBR Premix Ex Taq II and 0.4 μM of each primer. The protocol included an initial denaturation step at 95 °C for 15 min, followed by 32 cycles of 95 °C for 20 s, 60–65 °C for 30 s and 72 °C for 30 s. After amplification, a melting curve was obtained by increasing temperatures from 65 °C to 95 °C with fluorescence detection at 0.2 °C intervals.

The quantification of the target gene was performed using the cycle threshold (Ct) value in a PCR amplification curve by cluster analysis with variable cluster endpoints. Data were determined from duplicate assays. For normalization, the cell number in the specimen was determined from glyceraldehyde 3-phosphate dehydrogenase (GAPDH) gene quantification.

### 2.5. siRNA and miRNA Printing

siRNA/miRNA transfection solution was prepared essentially as described [38,39,40,41]. Briefly, 2 μL of 20 μM siRNA was transferred into 384 well plates. 6 μL of Red mixture, which contains 20 μM RED siGLO (Thermofisher, West Lafayette, CO, USA) 2 μL of 0.8 M sucrose dissolved in OptiMEM media and 2 μL of RNASE free water were added. Then, lipofectamine 2000 was added and mixed thoroughly. Then, the mixture was incubated for 20 min at RT, and 5 μL of 0.3% (w/v) gelatin was added. The final transfection reagent mixture was dispensed or printed as 300 µm spots on a microscope glass slide (MAS slides, Mutsunami, Japan) using stealth pins SMP 9 (Telechem, Sunnyvale, CA, USA) and a high throughput microarray printer (Genomic Solutions, Ann Arbor, MI, USA) at 22–25 °C, 55–65% RH (room humidity) enclosed in a custom built clean chamber providing a sterile HEPA filtered atmosphere. For some experiments with siRNA, we also tested glass cover slips (#1 Marienfeld, Germany) coated with poly-L-lysine (Sigma, St. Louis, MO, USA). Arrays were stored in a desiccator with no significant alterations in performance from 1 week to 5 months post-printing. Five slides covered the genome and contained 16% of control siRNA. We printed the genomic library such that the whole library was composed of 5-arrays set with 3,888 siRNA spots per array, containing 4 different sequences for each gene of the 18,000 individual human open reading frames from the Dharmacon “On-TARGET *plus* siRNA” library.

### 2.6. Microarray-Based Phenotypic Assay and Data Analysis

Cells were seeded at 2 × 10^6^ cells on microarrays located in 4-well cell culture dishes (Nunc, Drive Rochester, NY, USA) and cultured for 48 h to allow the transient transfection to occur. Cells were fixed and stained with anti-RelA antibody, as described for the well-based assay (see Section 2.3). Confocal images were acquired 48 h post-transfection using ImageXpress Ultra (Molecular Devices), 10× objective lenses. Three channels were used for the readout of RelA expression at 488 nm (FITC), for the detection of siGLO-RED at 560 nm (Texas Red) and for the detection of nucleus staining with DRAQ 5 at 635 nm. Quantification of p65 silencing was performed using MetaXpress software (Molecular Devices). First, we extracted red miRNA spots based on spot intensity at 560 nm. Only those cells that were associated with that spot area were analyzed to measure RelA expression. The intensity of the RelA signal/pixel was thresholded to remove cells with a low intensity of RelA signal from the analysis. The results were normalized to the total amount of cells in the image and converted in the percent of expressing cells (or % of expression), as described for image analysis of the cells on plates (Section 2.3).

## 3. Results and Discussion

### 3.1. Phenotypic Assay of siRNA and miRNA Knock-down Effect in Cellular Model

**Figure 1:**
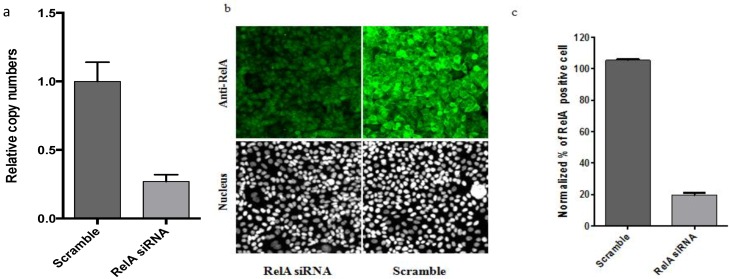
Direct transfection of Hela cells with RelA siRNA. Hela cells were transfected with RelA targeted siRNA *vs*. scramble siRNA for 48 h. (**a**) The level of RelA mRNA was measured by qPCR after transfection of anti-p65 siRNA and incubation for 48 h. (**b**) Cells transfected with anti-p65 siRNA were stained with anti-RelA antibody (Green) and DRAQ5 for nuclei detection (white (nucleus)). The exposure for the representative cell images for this figure was increased to show the presence of the cells in the images where the signal would be too low. (**c**) Image analysis used MDS MetaMorph image analysis software. The total level of RelA signal was analyzed as described in Section 2.3. The resulted data are presented as % of expressing cells (RelA positive cells). Data are plotted as bar-graphs using GraphPad Prism software, the error-bar representing the standard deviation for n = 3 replicates.

First, we established the functional assay that allows for the quantification of the cellular response to the transfected RNAi. As mentioned above, we chose to analyze the changes in the endogenous level of NF-κB protein after transfection with siRNA or miRNA. For the siRNA experiment, we selected OTP smart pool siRNA designed against the RelA member of the NF-κB family and, as a negative control, the OTP non-targeted siRNA #1. As any pooled siRNA, this sample consists of four different siRNA sequences against RelA to increase the silencing efficiency and decrease the chance of off-target effects. As was mentioned in the introduction, several miRNAs were found to control NF-κB expression. We selected has-miR-373 and has-miR-520c-3p mimic, as a miRNA that targets NF-κB, and microRNA mimic negative control #1 from the human miRIDIAN microRNA collection from Thermo scientific. The basic conditions that we are carrying out in the lab for direct transfection (see Experimental Methods) allow us to achieve 80% reduction of the mRNA copy number based on the RT-PCR analysis (Figure 1(a)). The same transfection protocol was used for image analysis of the cells in the treated wells. Figure 1(b,c) shows that the image analysis detecting anti-p65 (RelA) staining yielded a similar ~80% reduction of the RelA protein level after transfection of the anti-RelA siRNA. 

**Figure 2:**
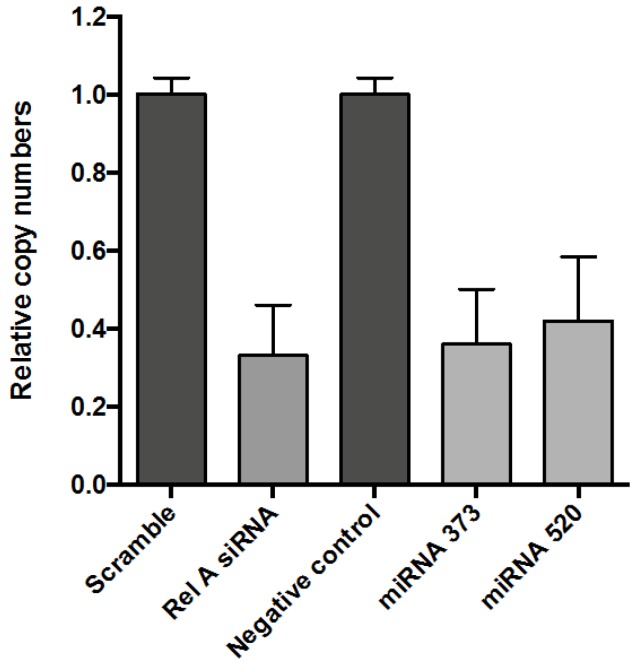
Comparison of NF-κB expression modulated by siRNA and miRNA-373c, miRNA-520 transfection. Hela cells were transfected with 20 nM of scramble siRNA and anti-RelA SiRNA, and the negative control (NC) for microRNA, miR-373c and miR-520 and incubated for 48 h before harvesting and analyzing the level of endogenous p65 by real-time PCR.

Next, the miR-520 and miR-373c were transiently transfected in Hela cells following exactly the same protocol as for siRNA. As was mentioned before, the siRNA was a pool of four different sequences targeting the same mRNA, but miRNA was represented only by one individual sequence and might be less efficient in silencing the target. Figure 2 shows that both microRNAs, targeting 3'UTR of ReLA, gave a very similar reduction effect on the level of mRNA as the pooled siRNA targeting the mRNA directly.

**Figure 3:**
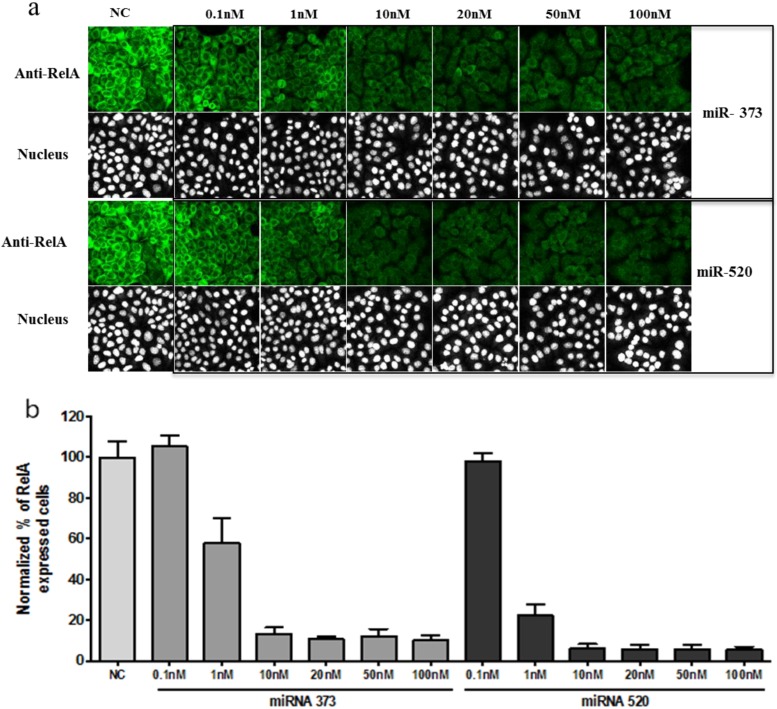
NF-κB expression was modulated by miRNA-373c and miRNA-520 transfection. Hela cells were transfected with negative control (NC), miR-373c and miR-520 and incubated for 48 h before fixation and image analysis. (**a**) Cells were stained with anti-RelA antibody (Green) and DRAQ5 for nuclei detection (white (nucleus)). As for the figure above, the exposure for cell images was increased to show the presence of the cells in the images where the signal would be too low. (**b**) Image analysis was carried out using MDS MetaXpress image analysis software. The RelA signal analysis is described in Section 2.3 and was presented as % of expressing cells (or % RelA-expressing cells). Data were plotted as bar-graphs using GraphPad Prism software, the error-bar representing the standard deviation for n = 3 replicates.

We performed the titration microRNA to be sure that the concentration of miRNA was optimal for efficient knock-down. The siRNA concentration that produces 80% knock-down usually varied around 20–50 nM; the miRNA produced a similar silencing effect, already at 10 nM (Figure 3), and 80% of silencing was detected as low as 1 nM for miR-520.

This is the first phenotypic approach demonstrating the down regulation of endogenous RelA protein in Hela cells in response to transfected miR-520 and miR-373c. This is also a proof of concept that the same phenotypic imaging approach can be used to detect the effect of both siRNA and miRNA on the endogenous level of NF-κB expression in cultured cells.

### 3.2. RNA Interference Microarrays

A robust high throughput approach is required to screen large libraries of RNAi designed for more than 18,000 individual genes of the human genome, as well as thousands of miRNAs or miRNA mimics designed by nature (or scientists) to control expression of those genes. Hybridization microarrays were mentioned above and currently used very broadly to study expression level in selected tissues or cells. We have adapted the high density spot microarrays for reverse-transfection of RNAi or cDNA into cells with microscopic monitoring of the changes in the selected phenotype or biomarkers. In our next experiments, we adapted the phenotypic analysis of RelA regulation by siRNA and micro-RNA to microarray-based reverse-transfection assay.

#### 3.2.1. High-Density Spot Microarrays for Reverse Transfection

**Figure 4:**
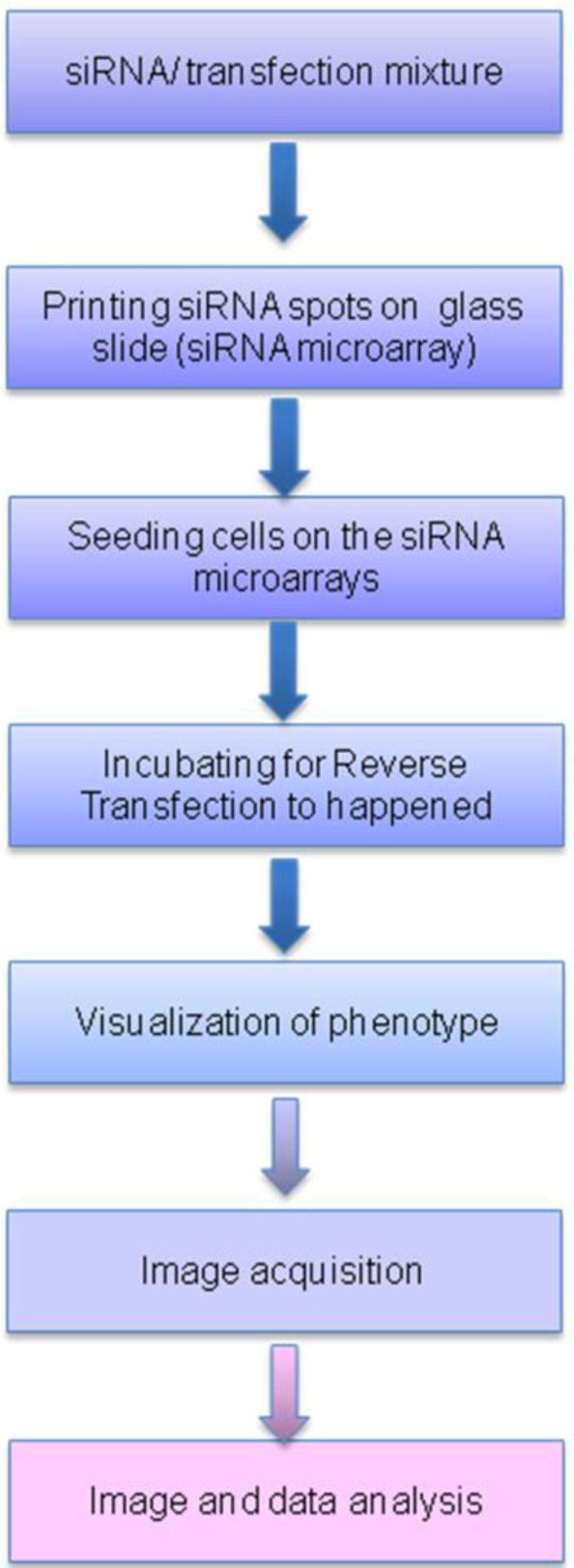
The flow chart for reverse-transfection of RNAi into cell monolayers on microarrays.

The steps for array-based reverse-transfection (Figure 4) are different from those of the forward transfection in wells described in Section 2.2. 

One of the main issues for reverse transfection is that the RNAi should be immobilized with a transfection reagent on the glass-slide in the form of a spot with a defined size and location. Once the cells are seeded on the top of the spots, RNAi should be transferred into cells attached right above the spot. To print uniform spots with a defined size, to reach high efficiency of transfection and to prevent cross contamination between spots, those are critical aspects in the process of the protocol optimization. The reverse transfection into cultured cell monolayers was fist demonstrated for cDNA constructs transfected into the Hek293 cell line by Ziauddin, J. and Sabatini in 2001 [21] and later was applied to the screening of a subset of siRNA libraries [22,23,38].

To adapt this method for experimental use, we applied multistep optimization to achieve accurate dispensing of the transfection mixture on the glass slides and efficient reverse transfection of the recombinant molecules. Several parameters needed to be considered simultaneously:

**A.** Selection of the right coating of the glassware that would efficiently immobilize RNAi reagents in a spot, preventing cross-contamination between spots and allow cell adherence on the slide and on the spot after seeding cells on the slide. We used poly-L-lysine and/or the MAS coating of the microarray glass slides. The majority of adherent cell lines that were tested in the lab demonstrated proper attachment to those surfaces. The printed microarrays can be stored at a low humidity environment (~10%) up to 12 months without the significant change of the transfection efficiency of the spotted reagent.

**B.** The transfection reagent mixture used for the arrays contains not only RNAi and the transfection reagent, but also labeled tracer RNA molecules for spot visualization (siGLO-RED in our case), gelatin and sucrose, which provide better immobilization of reagents after printing, as well as more efficient delivery of the RNA/transfection reagent complex into the cells. (see Table 1 and Section 2.2, Section 2.5). We optimized the composition of the reagent mixture that was used to print the OTP siRNA library (Table 1) and will discuss below the optimization of the reagent mixture for miRNA printing on glass slides. There is a big variety of commercially available transfection reagents that were developed to achieve good transfection efficiency with minimal cytotoxic effects. If there was a new cell line that needed to be transfected in microarray-based assays, we would have to test several of those reagents, like Lipofectamine 2000, RNAiMax and DharmaFECT, to obtain the best results.

**Table 1:** The composition of transfection reagent mixture for well-based or microarray-based transfection.

Component of mixture for reverse transfection					
in wells			on array		
**1**	lipofectamine	0.1 μL	**1**	lipofectamine	3 μL
	DMEM	9.9 uL			
**2**	SiRNA (1 μM)	5 μL (1 μM)	**2**	SiRNA (20 μM)	2 μL (20 μM)
	DMEM	5 μL	**3**	0.3 M Sucrose in OptiMEM	2 μL (0.3 M)
				RNAse free water	2 μL
Mixed and incubate for 20 min			Mixed and incubate for 20 min		
3	OptiMEM	80 μL	**4**	siGLO (20 μM)	2 μL
			**5**	0.2% gelatine	5 μL
add 100 μL to cells in 1 well/96 well plate or 20 μL in 1 well/384 well plate			Printed 3–6 μL/300 μm spot		

**Figure 5:**
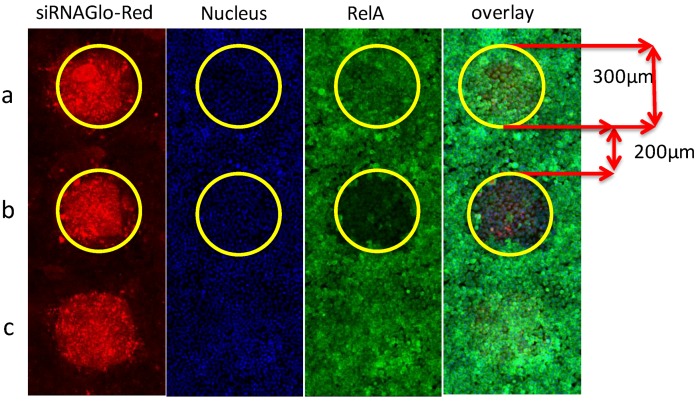
Spotting of siRNA on microarrays. Mixture of siRNA, siGLO (red) and transfection reagents described in Table 1 were printed on PLL-coated slides. The red is the detection of siGLO-RED siRNA tracer at the 560 nm channel for visualization of the printed spots. Cells were stained with an anti-RelA/secondary antibody labeled with Alexa 488 (green), detected at 488 nm channel, and DRAQ5 (blue), measured at 635 nm for nuclei detection. (**a**,**c**) Containing non-targeting siRNA; (**b**) anti-RelA siRNA; the reduction of green immuno-staining signals indicates the decrease of NF-κB protein expression in cells.

**Figure 6:**
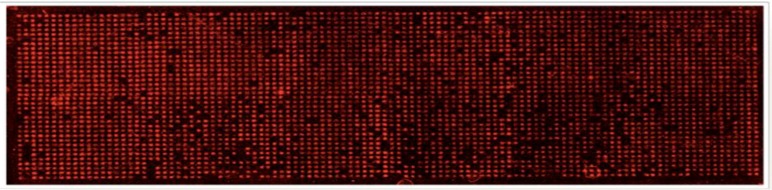
High density spot siRNA microarray. The tissue culture glass with siGLO siRNA tracer (red) spots visualized at 561 nm to assess the quality of the array printing.

**C.** The size and quality of the spots that reflect the amount of reagent that will be delivered into cells must be consistent through all arrays. It depends not only on the size of the pins selected for printing, but also on the viscosity of the transfection mixture, time and depth of the pin immerging in the reagent. With our selection of the pin-size, MAS coating for slides and composition of the siRNA transfection mixture, as listed in Table 1, the size of the spots was about 300 µm, with a distance between spots of ~200 µm (Figure 5). Those dimensions allowed us to print 4,000 spots containing individual siRNAs on one 26 × 76 mm glass slide (Figure 6). Thus, the whole library of 18,000 human genome siRNAs occupies only five slides. This miniaturization allows us to run multiple replicates during the genomic screen to ensure high quality results [40,41].

#### 3.2.2. Optimization of the Transfection Reaction for siRNA and miRNA on Microarrays

**Figure 7:**
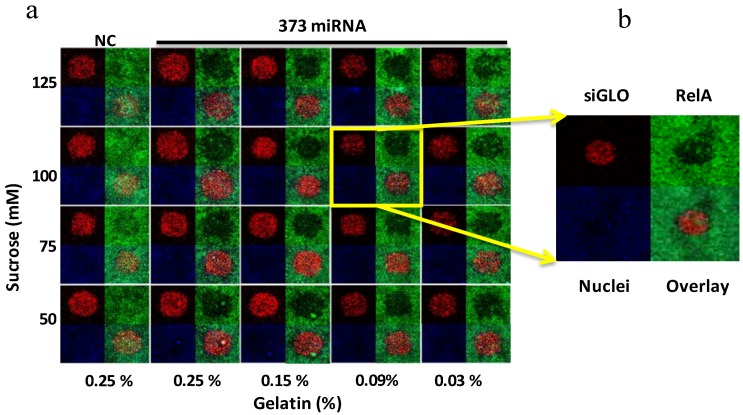
Optimization of the transfection reagent composition for miRNA array. (**a**) The immunostaining for RelA with Alexa 488 in Hela cells on small-scale siRNA microarrays containing miR-373 and non-targeted miRNA (NC, negative control) printed on MAS-coated glass slide with a matrix titration of different components of the transfection reagent mixture: sucrose from 50–125 mM (vertical) and gelatin from 0.03–0.25% (horizontal). (**b**) Annotation for the images of the cells representing three channels: siGLO-560 nm (Red), for spot localization; Nuclei-635nm (Blue), DRAQ5 signal; RelA-488 nm (Green). The overlay is the image created by merging all three images together.

The selected concentrations of sucrose and gelatin were also associated with the specific coating of the glassware used in this experiment. In order to immobilize miRNA spots for efficient delivery of miRNA into cells, both the gelatin and the sucrose are essential components in the miRNA transfection mixture. To optimize the miRNA reverse transfection efficiency, we tested gelatin and sucrose in a combination experiment. The high concentrations of both gelatin and sucrose were not optimal in spite of the better transfection efficiency, not only due to cross-contamination caused by the spread of the reagent form the spot area and inaccurate dispensing of the reagent, but because of the high viscosity of mixture. The results in Figure 7 show the matrix experiment designed to select the optimal concentrations of both reagents. The selected conditions, 100 mM sucrose and 0.09% gelatin, were confirmed in several experiments, not only with miR-373c, but also with miR-520 (data not shown).

**Figure 8:**
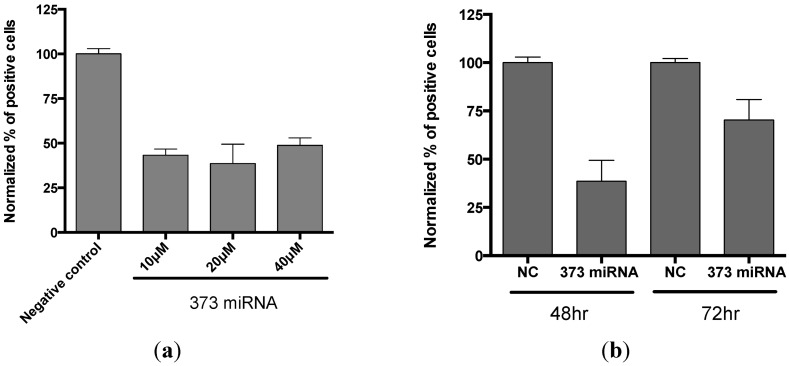
Optimization of the conditions for miRNA microarrays. (**a**) Test of different concentrations of miR-373 (373 miRNA) and non-targeted negative control in microarray. Data is plotted by GraphPad Prism, n = 4. (**b**) The time course for incubation, 48 and 72 h, of the cells on microarray. Data is plotted by GraphPad Prism; error bar: S.D., n = 4. All images of cells in experiments (a) and (b) were processed as described in Section 2.6.

When cells are transfected with siRNA or miRNA, the concentration of those molecules needs to be minimal to avoid any cytotoxic effects, but sufficient to produce a >70% knock-down effect on the targeted protein. The concentration of the siRNA stock that needs to be added to the reagent mixture was 10–20 uM. The range of concentrations was tested for miR-373 (Figure 8(a)). As the inhibitory RNA requires time to mediate the degradation of targeted mRNA and to decrease the overall protein level detected in cells, in our experiments, siRNA usually takes 48 to 72 h after transfection to establish a silencing effect for proteins like RelA. That effect starts to disappear after 96 h post-transfection (data not shown). We also performed a kinetic study for miRNA transfection. Figure 8(b) demonstrates that the effect of the miRNA-373 diminishes slightly after 72 h. Thus, the 48 h incubation time appears to be optimal for both siRNA and miRNA.

### 3.3. Phenotypic Assay for Cellular Monolayer Microarrays with miRNA and siRNA

**Figure 9:**
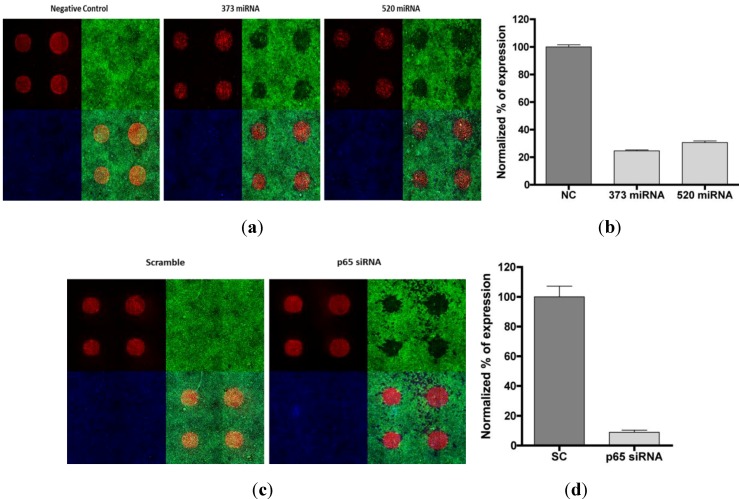
miRNA high density spots microarray. (**a**) The array of miRNA (miR-373 and miR-520 and non-coding control) spots used for phenotypic assay and quantitative image analysis: each test (square) shows three images from three channels (red: spots; green: RelA signal; blue: nuclei) and one overlay/merge of those three images (see annotation for Figure 6(b)). (**b**) The bar-graph plot of the data from images normalized as described in methods and presenting the % of cells expressing a detectable level of NF-κB. The data shows the average % of cells expressing RelA, n = 4; error: S.D. In a Student’s *t* test, the difference between the control and each of the miRNAs is significant, with *p* < 0.0001. (**c**) The array of siRNA (anti-RelA and scramble non-targetd control) spots for phenotypic assay and quantitative image analysis. (**d**) The bar-graph plot of the data from images for siRNA spots. *t* test: *p* < 0.0001.

As shown in Figure 1, Figure 3, both anti-RelA siRNA and miR-520/373 produced endogenous RelA protein knock-down in Hela cells. We applied the phenotypic assay using high content image analysis to the cells seeded and cultured on microarrays. The conditions for reverse transfection were optimized for both types of inhibitory RNA molecules. Cells were incubated on microarrays for 48 h to maximize the silencing of the RelA detected by imaging. Figure 9 shows that the knock-down effect produced by the siRNA and the miRNA is significant and comparable. However, the effect of miR-520 was slightly lower than expected, but still showed >70% silencing efficiency of the total RelA detectable in the cells.

### 3.4. Discussion

The goal of this work was to adapt the previously used phenotypic siRNA microarray-based screening methodology [40,41] to screening of miRNAs, a new group of RNA interfering molecules. Our phenotypic approach is using analysis of cellular images acquired by high resolution confocal microscopy to assess the changes of biomarkers or proteins of interest, as well as other morphological changes in a variety of cells, including immortalized cell lines, primary cells or stem cells. The use of the endogenous cellular environment is a critical aspect that makes phenotypic assays more biologically relevant and better suited to study the mechanisms that are taking place in tissues and even in whole organs. The application of phenotypic screening could be used in a variety of projects to search for new regulatory (like miRNAs) or functional components that play a crucial role in different diseases, like tumorigenesis, liver, kidney, cardio- or autoimmune disorders [14,17,42,43].

In this work, we have developed the first phenotypic cell-based microarrays for screening of the miRNA or synthetic miRNA mimic collections. Phenotypic microarray screening (or PhenomicID™) allowed us to perform thousands of individual experiments under controlled conditions to analyze large RNAi collections. The uniformity of the conditions ensures the reproducibility and the statistical reliability of individual observations. This feature makes the microarray approach a reliable tool for complex phenotypic assays [40,41]. Another advantage of microarray-based techniques is the significant cost reduction, due to the miniaturization of the volumes of reagents used for array printing (pmoles of materials and pL of volumes) and for the detection assays. Of course, there is a primary investment in building up the printing capabilities in the laboratory that need to be considered. The third, but not less critical, advantage is the significant increase of the throughput that is only comparable to the use of 1,536 and 3,456-well plate formats. The microarrays characteristics allow very complex experimental designs with multiple replicates and a large variety of samples. We have been able to apply the microarray screening of siRNAs in combination with six different concentrations of a compound of interest, which altogether used 21 copies of the human genome siRNA library (results from the laboratory in preparation for publication). As we have demonstrated in this work, the phenotypic microarray technology fits perfectly well to analyze the functions of miRNA collections in which each molecule could potentially control hundreds of genes and produce a clear phenotypic effect on cellular functions critical in development and disease. For example, a large set of miRNAs are known to be under-expressed in human tumors compared to normal tissue [44]. That analysis was done using hybridization techniques. The introduction of the same panel of microRNA mimics into relevant cell lines via microarray-based transfection would potentially result in reduction of cell proliferation and initiation of differentiation or apoptotic phenotypes detected by phenotypic analysis [45]. Such cell line profiles could add new specific microRNAs to anti-oncogenic or anti-metastatic pathways. It could be even more interesting to test the anti-miRNA molecules as a potential suppressors of miRNAs overexpressed in some diseases, such as autoimmune deregulations in lupus models [46]. 

## 4. Conclusions

Regulation of the NF-κB complex transcription and activation is very critical for cellular functions and, overall, cell fate. In this work, phenotypic cellular imaging was used to assess the effect of miRNAs, in particular, miR-520/373, on the total level of RelA protein detectable in Hela cells. The combination of phenotypic assays with high-density spot microarrays will allow for the screening of miRNA libraries in order to identify new molecules involved in the control of NF-κB or other critical cellular factors.

## References

[1] Fire A. (1999). RNA-triggered gene silencing. Trend Genet.

[2] Sharp P.A. (2001). RNA interference—2001. Genes Dev..

[3] Elbashir S.M., Harborth J., Lendeckel W., Yalch A., Weber K., Tuschl T. (2001). Duplex of 21-nucleotide RNAs mediates RNA interference in cultured mammalian cells. Nature.

[4] Wianny F., Zrnicka-Goetz M. (2000). Specific interference with gene function by double-stranded RNA in early mouse development. Nature Cell Biol..

[5] Sharma S., Rao A. (2009). RNAi screening: Tips and techniques. Nat. Immunol..

[6] Seyhan A.A., Ryan T.E. (2010). RNAi screening for the discovery of novel modulators of human diseases. Curr. Pharmaceut. Biotechnol..

[7] Mohr S., Bakal C., Perrimon N. (2010). Genomic screening with RNAi: Results and challenges. Ann. Rev. Biochem..

[8] Sood P., Krek A., Zavolan M., Macino G., Rajewsky N. (2006). Cell-type-specific signatures of microRNAs on target mRNA expression. Proc. Natl. Acad. Sci. USA.

[9] Baek D., Villen J., Shin C., Camargo F.D., Gygi S.P., Bartel D.P. (2008). The impact of microRNAs on protein output. Nature.

[10] Guo H., Ingolia N.T. (2010). Mammalian microRNAs predominantly act to decrease target mRNA level. Nature.

[11] Pasquinelli A.E., Hunter S., Bracht J. (2005). MicroRNAs: A developing story. Curr. Opin. Genet. Dev..

[12] Janas M.M., Wang E., Love T., Harris A.S., Stevenson K., Semmelmann K., Shaffer J.M., Chen P.H., Novina C.D. (2012). Reduced expression of ribosomal proteins relieves micro-RNA-mediate repression. Mol. Cell.

[13] Wang Z. (2011). The guideline of the design and validation of miRNA mimics. Methods Mol. Biol..

[14] Sokilde R., Barken K.B., Mouritzen P., Moller S., Litman T., Gusev Y. (2010). MicroRNA expression analysis by LNA enhanced microarrays. MicroRNA Profiling in Cancer.

[15] Pandey P., Brors B., Srivastava P.K., Bott A., Boehn S.N.E., Groene H.J., Gretz N. (2008). Microarrays-based approach identifies microRNAs and their target functional patterns in polycystic kidney disease. BMC Genomics.

[16] Keklikoglou I., Koerner C., Schmidt C., Zhang J.D., Heckmann D., Shavinskaya A., Allgayer H., Guckel B., Fehm T., Schneewewiss A., Sahin O., Wiemann S., Tschulena U. (2011). MicroRNA-520/373 family functions as a tumor suppressor in estrogen receptor negative breast cancer by targeting NF-κB and TGF-β signaling pathways. Oncogene.

[17] Zhang Y., Fan K.J., Sun Q., Chen A.Z., Shen W.L., Zhao Z.H., Zheng X.F., Yang X. (2012). Functional screening for miRNAs targeting Smad4 identified miR-199a as a negative regulator of TGF-β signaling pathway. Nucl. Acids Res..

[18] Eulalio A., Mank M., Ferro M.D., Zentilin L., Sinagra G., Zacchigna S., Giacca M. (2012). Functional screening identifies miRNAs inducing cardiac regeneration. Nature.

[19] Schena M., Shalon D., Davis R.W., Brown P.O. (1995). Quantitative monitoring of gene expression patterns with a complementary DNA microarray. Science.

[20] Cheung V.G., Morley M., Aguilar F., Massimi A., Kucherlapati R., Childs G. Making and Reading Microarrays. http://www.rose-hulman.edu/~ahmed/making%20and%20reading%20cdna%20microarrays.pdf.

[21] Barbulovic-Nad I., Lucente M., Sun Y., Zhang M., Wheeler A.R., Bussmann M. (2006). Bio-microarray fabrication techniques—A review. Crit. Rev. Biotechnol..

[22] Ziauddin J., Sabatini D.M. (2001). Micro-array of cells expressing defined cDNAs. Nature.

[23] Silva J.M., Mizuno H., Brady A., Lucito R., Hannon G.J. (2004). RNA interference microarrays: High-throughput loss-of-function genetics in mammalian cells. PNAS.

[24] Mousses S., Caplen N.J., Cornelison R., Weaver D., Basik M., Hautaniemi S., Elkahloun A.G., Lotufo R.A., Choudary A., Dougherty E.R., Suh E., Kallioniemi O. (2007). RNAi microarrays analysis in cultured mammalian cells. Genome Res..

[25] Karin M., Greten F.R. (2005). NF-κB: Linking inflammation and immunity to cancer development and progression. Nat. Rev. Immunol..

[26] Karin M. Nuclear factor-κB in cancer development and progression. Nature.

[27] Hoffmann A., Baltimore D. (2006). Circuitry of nuclear factor κB signaling. Immunol. Rev..

[28] Oeckinghaus A., Ghosh S. (2009). The NF-κB family of transcription factors and its regulation. Cold Spring Harb. Perspct. Biol..

[29] O’Donnell K.A., Wentzel E.A. (2005). c-Myc-regulated microRNAs modulate E2F1 expression. Nature.

[30] He L., Thomson J.M., Hemann M.T., Hernando-Monge E., Mu D., Goodson S., Powers S., Cordon-Cardo C., Lowe S.W., Hannon G.J., Hammond S.M. (2005). A microRNA polycistron as a potential human oncogene. Nature.

[31] Mraz M., Pospisilova S., Malinova K., Slapak I., Mayer J. (2009). MicroRNAs in chronic lymphocytic leukemia pathogenesis and disease subtypes. Leuk. Lymphoma.

[32] Cordes K., Srivastava D. (2009). MicroRNA regulation of cardiovascular development. Circ. Res..

[33] Ma X., Becker Buscaglia L.E, Barker J.R., Li Y. (2011). MicroRNAs in NF-κB signaling. J. Mol. Cell Biol..

[34] Liu P., Wilson M.J. (2012). MiR-520c and miR373 upregulate MMP9 expression by targeting mTOR and SIRT1, and activate the Ras/Raf/MEK/Erk signaling pathway and NF-κB factor in Human fibrosarcoma cells. J. Cell. Physiol..

[35] Wang L., Kang F., Shan B., Liu L., Sang M. (2012). Targeting NF-κB p65 with an artificial microRNA suppress growth of MDA-MB-231 human triple-negative breast cancer cell line. Gene Ther. Mol. Biol..

[36] Huang S., Robinson J.B., Deguzman A., Bucana C.D., Fidler I.J. (2000). Blockade of nuclear factor-κB signaling inhibits angiogenesis and tumorigenecity of human ovarian cancer cells by suppressing expression of vascular endothelial growth factor and interleukin 8. Cancer Res..

[37] Huang Q., Gumireddy K., Schrier M., le Sage C., Nagel R., Nair S., Egan D.A., Li A., Huang G., Pure E., Agami R. (2008). The microRNAs miR-373 and miR-520c promote tumor invasion and metastasis. Nat. Cell Biol..

[38] Erfle H., Neumann B., Liebel U., Rogers P., Held M., Walter T., Ellenberg J., Pepperkok R. (2007). Reverse transfection on cell arreys for high content screening microscopy. Nat. Protocol..

[39] Erfle H., Neumann B., Rogers P., Bulkescher J., Ellenberg J., Pepperkok R. (2008). Work flow for multiplexing siRNA assays by solid-pahse reverse trasnfection in multiwell plates. J. Biomol. Screen..

[40] Genovesio A., Giardini M.A., Kwon Y.J., Dossin F.D.M., Choi S.Y., Kim N.Y., Kim H.C., Jung S.Y., Schenkman S., Almeida I.C., Emans N., Freitas-Junior L.H.F. (2011). Visual genome-wide RNAi screening to identify human hostfactors requered for *Trypanosoma cruzi* infection. PLoS One.

[41] Genovesio A., Kwon Y.J., Windisch M.P., Kim N.Y., Choi S.Y., Kim H.C., Jung S., Mammano F., Perrin V., Boese A.S., Casartelli N., Swartz O., Nehrbass U., Emans N. (2011). Automated genome-wide visual profiling of cellular proteins involved in HIV infection. J. Biomol. Screen..

[42] Ikeda S., Kong S.W., Lu J., Bisping E., Zhang H., Allen P.D., Golub T.R., Pieske B., Pu W.T. (2007). Altered microRNA expression in human heart disease. Physiol. Genom..

[43] Zhu S., Pan W., Sing X., Liu Y., Tang Y., Liang D., He D., Wang H., Liu W., Shi Y., Harley J.B., Shen N., Qian Y. (2012). The microRNA mir-23b suppress IL-17-associated autoimmune inflammation by Targeting TAB2, TAB3 and IKK-α. Nat. Med..

[44] Lu J., Getz G., Miska E.A., Alvarez-Saavedra E., Lamb J., Peck D., Sweet-Cordero A., Ebert B.L., Mak R.H., Ferrando A.A., Downing J.R., Jacks T., Horvitz H.R., Golub T.R.  (2005). MicroRNA expression profiles classify human cancers. Nature.

[45] Tavazoie S.F., Alarcon C., Oskarsson T., Padua D., Wang Q., Bos P.D., Gerald W.L., Massague J. (2008). Endogenous human microRNAs that suppress breast cancer metastasis. Nature.

[46] Dai R., Zhang Y., Khan D., Heid B., Caudell D., Crasta O., Ahmed S.A. (2010). Identification of a common lupus disease-associated microRNA expression pattern in three different murine models of Lupus. PLoS One.

